# An ecological examination of the emotions of Chinese high school mathematics teachers in a long-term teaching improvement program

**DOI:** 10.3389/fpsyg.2022.1033988

**Published:** 2022-12-19

**Authors:** Peiyao Lei, Su Han, Wenqing Kong, Sunzhong Lv, Xiaoqin Wang

**Affiliations:** ^1^School of Mathematical Sciences, East China Normal University, Shanghai, China; ^2^College of Teacher Education, Faculty of Education, East China Normal University, Shanghai, China

**Keywords:** teacher emotion, ecological framework, teacher professional development, mathematics teacher, case study

## Abstract

Teacher emotions are essential for teaching effectiveness and teachers’ professional development. Studying teacher emotions during a program in today’s world is crucial, where teachers are commonly involved in professional development programs. From an ecological perspective, this study employed a case study method to examine the emotions of two Chinese high school mathematics teachers in a 4-year teaching improvement program. Semi-structured interviews, teacher emotion logs, researcher memos, and informal dialogues were all used to collect data. This study aimed to better understand the emotions that Chinese high school mathematics teachers developed and the processes that generated them in their interactions with various ecosystems within a specific professional development program overlay. The study’s findings revealed that the two teachers triggered 65 emotions in their interactions with the various ecosystems over 4 years—A describing 51 and B describing 46—with an overall predominance of positive emotions. They triggered the broadest range of emotions in the mesosystem, particularly during interactions with program companions. During the early, medium, and late stages, their internal psychological factors and interactions with each system changed, resulting in significant changes in their emotions. They all went through phases of mixed sadness and happiness, ending in a state of delight and calmness. Finally, we recommended teachers’ professional development based on the study’s findings.

## Introduction

Teacher emotions have emerged as an important research topic in psychology and education, and they are critical for teacher’s identity, teaching quality, teacher–student relationships, student performance, and even educational reform (Hargreaves, 2005; Frenzel et al., 2009a, 2020; Schutz and Zembylas, 2009; Kunter et al., 2011). Therefore, researchers all over the world are interested in teachers’ emotions. They have investigated various categories, antecedents, and consequences of teacher emotions (Frenzel et al., 2009b; Bahia et al., 2013; Aldrup et al., 2017; Taxer et al., 2019; Buric et al., 2020; Martínez-Sierra et al., 2022; Panadero et al., 2022; Tao, 2022). As research on teacher emotions has advanced and deepened, researchers have realized that teachers develop emotions within person–event interactions under different environments (Schutz et al., 2006). Rather than simple intrapersonal feelings, teachers’ emotions are rooted in a complex ecology. An ecological perspective, free of the constraints of single-factor thinking, enables an integrated and systematic analysis of the complex interactions between teachers and their environments (Gu and Gu, 2015). This research perspective is emerging in teacher emotion research (Cross and Hong, 2012; Chen, 2020; Sun and Yang, 2021).

Research has revealed that teacher emotions differ across disciplines and key stages in school education (such as primary, middle, high school, and university; Frenzel et al., 2009b, 2015). Among the available studies, research on language teachers dominates the discipline research (Golombek and Doran, 2014; Chu et al., 2021; Heydarnejad et al., 2021; Sun and Yang, 2021; Liu et al., 2022), and the research on key stages is mainly focused on primary school and university teachers (Gu, 2016; Chen, 2017, 2019; Lu and Zhang, 2021), whereas high school mathematics teacher emotions have received less attention (Martínez-Sierra et al., 2019; Jiang et al., 2021). However, the uniqueness of high school mathematics teachers in terms of their key stages and disciplines, particularly in China, where high school mathematics teachers are also under the pressure of *Gaokao* (the National College Entrance Examinations; Jiang et al., 2022), makes their professional background and emotional development unique and deserving of more in-depth and focused research.

In addition, many scholars have researched teacher professional development (hereafter referred to as PD) programs when such participation is widespread (e.g., Borko, 2004; van Es, 2012; Alles et al., 2019; Cai and Hwang, 2021). Although some studies have examined teacher emotions in PD programs, they have less frequently distinguished between teacher emotions in everyday teaching and PD programs (e.g., Sun and Yang, 2021). This distinction is necessary from a research perspective. Because teacher identity develops after participating in a PD program, shifting from custodians or imparters of existing knowledge to learners of content knowledge or pedagogical knowledge that PD points to, the disruption and reconstruction of identity bring new emotions (Kleinknecht and Schneider, 2013; Chang et al., 2018), such as vulnerability (Yoo and Carter, 2017). Furthermore, in PD programs, teachers’ interactions with mentors and companions are also more different from those of colleagues and administrators at the school level; rich and particular emotions can arise from these interactions (Borko et al., 2008; Kleinknecht and Schneider, 2013; Chang et al., 2018; Weddle et al., 2019).

Therefore, due to the lack of attention paid to the emotions of high school mathematics teachers, especially from an ecological perspective, the emotions of mathematics teachers in a PD program are studied in this paper. This research pays attention to the emotions of high school mathematics teachers participating in a four-year PD program. Bronfenbrenner’s ecological systems theory (Bronfenbrenner, 2005) has been applied to analyze the emotions developed by teachers as a result of their complex interactions with various levels of the environment throughout the program. More specifically, we hope to comprehend the complex emotions of high school mathematics teachers when confronted with various objects and events in a specific PD program overlay and the mechanisms that cause them. This will help us think about how to increase positive teacher emotions to facilitate their PD and provide more experiences and insights for relevant teacher education, researchers, and administrators.

## Literature review

### Teacher emotions

Since the turn of the last century, ‘emotion’ has been a significant topic in educational research (Hargreaves, 1998). From physiological and psychological perspectives, emotions are mostly regarded as individual psychological phenomena involving physical and psychological changes. However, from a sociological perspective, emotions are seen as socio-constructed, experienced, generated, and expressed by individuals in the context of their organizational, social, cultural, historical, and political relationships (Hargreaves, 1998; Sutton and Wheatley, 2003). Because education occurs in real and complex arenas, definitions of emotions in pedagogy are often a blend of definitions from multiple disciplines. For example, Schutz et al. (2006) contended that “Emotions in education are socially constructed, and personally enacted.” Since the teacher is one of the two main subjects of teaching and learning, scholarly attention to teacher emotions is growing. The research has concentrated on the categories, antecedents, and consequences of their emotions. In terms of categories, many studies categorize teacher emotions using psychological methods, such as dichotomous, multiple classifications, and dimensional analysis (e.g., Bahia et al., 2013; Taxer et al., 2019; Sun and Yang, 2021). For example, some studies classified teachers’ emotions into positive and negative emotions and discovered that teachers experienced more positive emotions than negative emotions in their careers, with the primary positive emotions being enjoyment, joy, love, and so on, and the primary negative emotions being anxiety, anger, and so on (Bahia et al., 2013; Frenzel et al., 2015; Taxer et al., 2019). In terms of antecedents, numerous studies have examined why teacher emotions develop from various perspectives. For example, Martínez-Sierra et al. (2022) investigated situations that triggered a specific teacher emotion, whereas Cross and Hong (2012) considered teacher emotions as a result of individual interactions with various ecological environments. Among them, Chen (2021) utilized the cutting-edge Preferred Reporting Items for Systematic Review and Meta-Analysis method to examine how teacher emotions were influenced by personal, contextual, and emotional capacity, with personal factors including knowledge, values, professional beliefs, and so on, contextual factors consist of policy, sociocultural, organizational, and stakeholder factors, and emotional capacity including emotional labor strategies, emotion regulation, and emotional intelligence. The findings provided a general overview for succeeding researchers to engage in teacher emotion research (Chen et al., 2022). Regarding consequences, teacher emotions can have a wide range of consequential effects (Hargreaves, 1998; Frenzel et al., 2009b; Chen, 2021). On the one hand, as teachers’ personal factors are not static but constantly reshaped, it is not difficult to imagine that teacher emotions will affect teachers first and foremost, such as teacher efficacy and professional identity in professional beliefs (Chen, 2021). Moreover, emotions influence teaching methods. For example, Chen (2019) discovered that positive teacher emotions, such as joy, could positively predict student-centered teaching methods, whereas negative emotions, such as sadness, could positively predict teacher-centered teaching methods. On the other hand, students, as the primary stakeholders that teachers face in their daily teaching, are affected not only by teacher emotions in the classroom (Frenzel et al., 2009a; Becker et al., 2014) but also have a far-reaching impact on their learning, including academic achievement (Kunter et al., 2011), motivation (Kunter et al., 2013), and so on. At the macro level, teachers’ emotions can influence the implementation of various educational reforms (Hargreaves, 1998; Lu and Zhang, 2021).

As teacher emotion research has advanced, many researchers have begun to detect and discover from comparative studies that teacher emotions reflect differences at different key stages in school education, as well as disciplines. In terms of the key stages in school education, Bahia et al.’s (2013) study found that teachers teaching at a higher school stage were more concerned with their personal emotions than teachers teaching at a lower school stage, whereas teachers teaching at a lower school stage were more concerned with their students’ emotional state. Other studies have discovered differences in teacher emotions at different key stages (Frenzel et al., 2009b). There are also differences among teachers who teach different disciplines (Becker et al., 2014; Frenzel et al., 2015). For example, for the three teaching-related emotions of enjoyment, anger, and anxiety, Frenzel et al. (2015) surveyed 135 German teachers who taught mathematics and science and found that their enjoyment and anger had apparent disciplinary specificity. Anxiety was less dependent on the discipline taught. Exploring the existing research further reveals an intriguing phenomenon: research on language teachers’ emotions has dominated teacher emotion research in recent years (Golombek and Doran, 2014), especially for English as a foreign language teachers (Chu et al., 2021; Shi, 2021; Sun and Yang, 2021; Liu et al., 2022), and the studies mentioned above have meticulously examined the rich types and antecedents of teacher emotions across language levels. In contrast, less research has been conducted on high school mathematics teachers’ emotions. Scholars have focused on the relationship between high school mathematics teachers’ emotions and classroom teaching in regular and innovative teaching (Martínez-Sierra et al., 2019; Jiang et al., 2021, 2022). These studies have typically collected data through questionnaires, classroom observations, interviews, and teacher self-reports, and the studies have ranged from individual cases to groups of teachers. For example, Jiang et al. (2021) investigated the relationship between teacher emotions and classroom teaching with a sample of 221 Chinese high school mathematics teachers. They concluded through confirmatory factor analysis and structural equation modeling that, compared to anger and anxiety, they experienced enjoyment and satisfaction more frequently, and different emotions were influenced by age, grade level, teaching experience, and professional title. Structural equation model (SEM) A showed that inquiry-based instruction positively predicted enjoyment and satisfaction and negatively predicted anger and anxiety, whereas direct instruction positively predicted anger and anxiety. SEM B showed that satisfaction could positively predict inquiry-based instruction.

Overall, these studies revealed the multifaceted, complex, and variable nature of teacher emotions in primary education and the focus and characteristics of research on teacher emotions. It is necessary and meaningful to conduct more focused research on teacher emotions in high school mathematics, considering the differences between disciplines and key stages in school education.

### Teacher emotions in professional development programs

For an increasing number of teachers, PD has become an essential part of their professional lives, and participation in a PD program within or outside of school is becoming more common (Borko, 2004; van Es, 2012). As PD research has grown, so has the content and form of PD programs (Borko, 2004). In terms of PD research, because PD emerged from researchers’ focus on the connections between teachers’ teaching practices and their knowledge, current PD research has a clear cognitive orientation (Tonga et al., 2022), with teacher emotions as a noncognitive aspect easily overlooked (Yoo and Carter, 2017). However, in studies of teacher emotions in PD programs, researchers have focused more on teacher emotions in their daily teaching (Cross and Hong, 2012; Cross et al., 2020) and less on the emotions triggered by the PD program itself. For example, Cross et al. (2020) studied the emotions of seven primary school teachers participating in the Holistic Individualized Coaching (HIC) improvement program over five rounds of the “Preparing to Teach–Implementing Teaching” cycle but did not focus specifically on the emotions caused by the HIC program itself. In the current research, teacher emotions in PD, particularly the unique emotions that arise from PD programs, have received little attention.

Previous research has focused on the emotions generated by teachers’ interactions with partners in PD (Borko et al., 2008; Kleinknecht and Schneider, 2013; Parr and Hawe, 2017; Chang et al., 2018), with the interactions primarily taking the form of video-based teaching observation, discussion, and reflection. For example, Kleinknecht and Schneider (2013) showed that others-viewing corresponds to a more profound analysis of problematic events and higher emotional engagement, such as disappointment and anger, in their study of five pairs of mathematics teachers independently analyzing teaching video processes in a web-based environment. The findings also revealed that self-viewing necessitates more pre-existing scaffolding than others-viewing. In contrast, the emotions generated by teacher–mentor interactions have piqued the interest of only a few scholars (Chang et al., 2018; Sun and Yang, 2021). Sun and Yang (2021) found in their study that teachers in PD felt confident when they received expert guidance and felt anxious when they believed they could not perform well. The emotions generated by teacher–mentor interactions are a field that warrants further research.

In addition, with the recent rise of lesson study (Huang and Shimizu, 2016), the teaching in PD programs has begun to shift from fragmented teaching and peer simulation teaching to realistically developing an exemplary lesson, which has been iteratively improved through the efforts of the entire lesson study group (Huang and Bao, 2006). In this context, it is worthwhile to investigate how teachers’ emotions in PD were overlaid in their daily teaching.

### An ecological perspective to examine teacher emotions

More and more research has suggested that teachers’ emotions are generated by interactions with people and events at various levels of the environment (Schutz et al., 2006). They are influenced not only by personal knowledge, beliefs, and emotion-related competencies (e.g., emotional intelligence and emotional regulation) but also by external factors ranging from students, colleagues, and parents to curriculum reform, educational change, and specific culture (Chen, 2021). As a result, researchers have widely recognized the complex ecology in which teachers’ emotions arise and develop, and an ecological perspective on teachers’ emotions is beginning to emerge.

At the beginning of this century, Zembylas (2007) proposed that “teacher knowledge is a form of knowledge ecology,” a system comprised of a symbiosis of multiple sources and forms of knowledge. Because emotion plays a massive role in the teaching and learning of teachers and students, teachers’ *emotional knowledge* is an inseparable part of their *knowledge ecology*, called *emotional ecology*. Zembylas (2007) constructed a system with a three-dimensional model of teacher emotion ecology with *individual plane*, *relational plane*, and *sociopolitical plane*. He applied this model to a case study of four preservice primary school teachers’ emotional ecology and pedagogical content knowledge (PCK). However, Bronfenbrenner’s (1995) ecological systems theory, which Zembylas references in his conceptual definition, has had a more academic impact on the field of teacher emotion research than Zembylas’s (2007) emotional ecology model for teachers.

Tissington (2008) applied Bronfenbrenner’s (1995) theory to conceptualize alternative certification teaching candidates. The theory’s four nested structures, including the microsystem, mesosystem, exosystem, and macrosystem, are specified as classroom practice, professional collaboration, organizational structure and policies, and cultural values, respectively. However, Tissington (2008) ignored the impact of continuous change in the environment in which people live over time on human development (i.e., the chronosystem), which Bronfenbrenner (1986) has long proposed, and Tissington’s (2008) study are not fully applicable to in-service teacher research. Since then, Cross and Hong (2012) expanded on Tissington’s (2008) research, drawing on Tissington’s perspective on individuals’ proximity to their environment. Moreover, they applied Bronfenbrenner’s ecological theory to teacher emotion research (Mohammadabadi et al., 2019; Bharaj and Singh, 2021; Carroll et al., 2021). Cross and Hong (2012) invited two mathematics teachers who worked in the same primary school serving a high-minority, high-poverty population as case studies. Interviews, classroom observations, and daily communications were conducted to investigate how their internal psychological characteristics interact with their external environment to produce emotions. According to their findings, interactions with students are the primary source of positive emotions. However, personal–environmental events trigger emotions like disappointment and frustration in all environments, from the microsystem (e.g., complacent colleagues) to the macrosystem (e.g., mandated testing), due to their positive educational beliefs and solid professional identities, they could carefully consider coping strategies to overcome negative emotions. It is also worth noting that the study ignored the chronosystem and did not address how interactions within the PD program influenced teacher emotions in the context of the two participants in the same three-year PD program (Cross and Hong, 2012).

Following Cross and Hong (2012), there has been a gradual increase in research on teacher emotions that references or directly applies Bronfenbrenner’s ecological systems theory, with research on language teachers still accounting for a large proportion of the field (Mercer, 2020; Chu et al., 2021; Hofstadler et al., 2021; Sun and Yang, 2021; Tao and Jiang, 2021; Liu et al., 2022; Wang, 2022). For example, Hofstadler et al. (2021) conducted semi-structured interviews with 16 teachers to explore how different ecological levels shape the professional subjective well-being of Content and Language Integrated Learning in Austria. The data analysis revealed that a complex set of nine factors, such as students, peer support, language-related issues, and so on, influence teachers’ subjective well-being across ecosystems’ personal, class, institutional, and national contexts. In addition to the positive emotion of well-being, Liu et al. (2022) explored teachers’ anxiety about online teaching in the context of the COVID-19 global pandemic through interviews with 12 Chinese high school English teachers, eventually identifying six types of anxiety from three systems: macrosystem, exosystem, and microsystem.

In terms of research methods, the majority of studies on teacher emotions that employed Bronfenbrenner’s ecological systems theory used qualitative research methods (Chu et al., 2021; Sun and Yang, 2021; Liu et al., 2022), with only a few utilizing mixed research methods. For example, Chen (2020) applied mixed methods to study Chinese primary school teachers from different disciplines. This study’s quantitative and qualitative data indicate that high-intensity positive and negative emotions exist in the microsystem and are primarily generated through interactions with students. Positive emotions include caring, happiness, and joy, while negative emotions include sadness, depression, and anger.

From the above literature review, it can be seen that examining teacher emotions from an ecological perspective is a relatively new development, but it has become a hot topic of research. Bronfenbrenner’s ecological systems theory has been widely used, allowing researchers to analyze and comprehend teacher emotions at different fine-grained levels. However, very few studies have examined the emotions of high school mathematics teachers in teaching improvement programs from an ecological perspective, and this study will attempt to do so.

### The present study

Through a review of research on teacher emotions, we found that the previous studies had following gaps: first, most of studies on teacher emotions had been focused on English teachers or elementary and college teachers, with fewer studies on high school mathematics teachers; second, most of studies on teacher emotions in PD programs had failed to distinguish the unique emotions that teachers generated in PD programs; finally, very little was known about the different types of emotions experienced by high school mathematics teachers in specific PD programs.

Based on above gaps, this paper aims to conduct an in-depth and detailed study of the emotions of high school mathematics teachers in a specific PD program, to achieve the following research objectives: (1) to identify the emotions experienced by high school mathematics teachers in a specific PD program; and (2) to depict how these emotions have emerged, especially through which interactions have these emotions been generated in the PD program.

Based on these two research purposes, we believe that the case study approach can assist us in gaining a deeper understanding of the development mechanisms of emotions of high school mathematics teachers in PD programs, because both these emotions and the emerging contexts are very complex, requiring rich data and careful analysis. Therefore, this research has been focused on two teachers in a four-year high school mathematics teaching improvement project, given that these two teachers had generated various emotions through the extended program participation. We expect that data analyzed from numerous sources for the two teachers will provide insight into what emotions high school mathematics teachers have experienced when they actively participate in a PD program over time and how these emotions have originated. This could demonstrate a model of emotional development for high school mathematics teachers under a specific PD project.

Specifically, this research hopes to address the following research questions:

1. What emotions did Chinese high school mathematics teachers develop when they interacted with each ecosystem during the teaching improvement program?
2. How did these emotions emerge?

## Analytical framework

Bronfenbrenner’s (2005) ecological framework focuses on the complexity of the person–environmental interaction from the individual’s sociocultural perspective. Cross and Hong (2012) utilized this ecological framework to analyze how teachers develop their individual emotions in their interaction with the environment. They contended that the human development environment was nested in layers consisting of the chronosystem, macrosystem, exosystem, mesosystem, and microsystem. Much prior research has demonstrated that an ecological framework is valuable for examining the contexts in which teachers exist and develop (Sun and Yang, 2021).

After the two teachers in this study joined the teaching improvement program, they interacted with different elements in different environments and identities. Their individual emotions were influenced by a complex mix of sociocultural and environmental factors. The ecological framework proposed by Bronfenbrenner (2005) was employed in this research to better understand teachers’ emotional development and the contexts in which they emerged as a result of multiple factors. Therefore, this framework was utilized to analyze the emotional development of two Chinese teachers while participating in a mathematics teaching improvement program.

**Figure 1:**
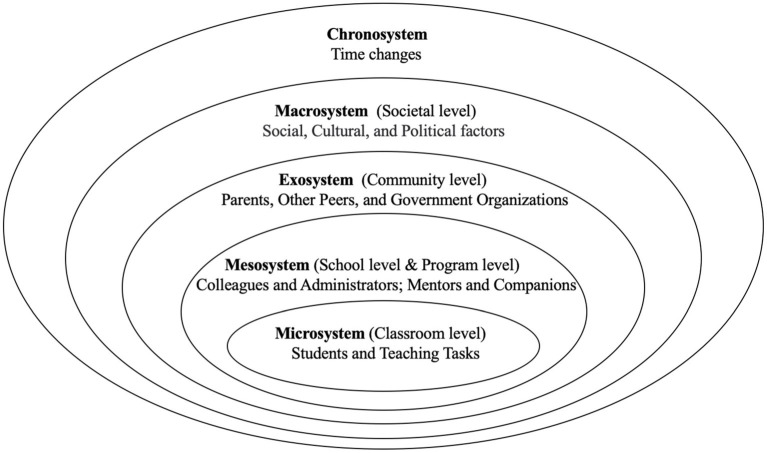
A revised framework adapted from Bronfenbrenner (2005) [as cited in Chen, 2020].

Within the ecological framework, teachers’ interactions with their environment span from close to far (Tissington, 2008; see Figure 1). The specific interactions between high school mathematics teachers’ emotions and the various systems were as follows.

The microsystem refers to “the structures and processes taking place in an immediate setting, containing the developing person (e.g., home, classroom, and playground)” (Bronfenbrenner, 2005, p. 80). As for the teacher’s ecological environment, classroom practices were at the innermost level of the framework, a pattern of activity and interaction within the teacher’s immediate environment (Tissington, 2008). For the teachers in this study, at the classroom level, where the program concepts were integrated, their interactions with students and the teaching task were included in this system.

The mesosystem includes connections with the microsystem (Bronfenbrenner, 2005, p. 80). In the teacher ecological environment, teachers frequently sought professional collaboration on a broader layer of the environment for development, which constituted a mesosystem (Tissington, 2008). In this study, teachers sought support primarily from schools and programs, and the mesosystem for teachers included links with colleagues and administrators at the school level and program mentors and program companions at the program level. Program mentors comprised the initiator and principal of the teaching improvement program, guiding teachers on teaching practice within the program concepts. Program companions included other people who communicated and collaborated with the two teachers in the program, including other teachers who carried out teaching practices and researchers who provided teaching materials.

The term “exosystem” refers to “the linkages and processes taking place between two or more settings, at least one of which does not ordinarily contain the developing person, but in which events occur that influence processes within the immediate setting that does contain that person” (Bronfenbrenner, 2005, p. 80). The exosystem, an extension of the mesosystem, is related to the larger community level. Teachers might or might not be directly tied to exosystem elements, but all of these elements influence teachers’ working lives (Chen, 2020). The exosystem for the teachers in this study was primarily comprised of parents, other peers, and government organizations. The other peers mainly refer to teachers from other schools who have no organizational relationship with the two teachers and are not involved in the program. Their interactions with the teachers were at the community level. Government organizations included the education administration in the local district government.

The macrosystem is defined as “an overarching pattern of ideology and organization of the social institutions common to a particular culture or subculture” (Bronfenbrenner, 2005, p. 81). The macrosystem reflected the broader societal level, with particular norms, values, regulations, and policies under which teachers implement their teaching tasks (Cross and Hong, 2012). The teachers’ working lives in this study were primarily influenced by social, cultural, and political elements, which were the most visible manifestations of the macrosystem.

The chronosystem permits “one to identify the impact of prior life events and experiences, singly or sequentially, on subsequent development” (Bronfenbrenner, 2005, p. 83). Teachers always interact with various students at different time slots. Changes in time inevitably result in changes in life events and experiences (Chen, 2020). As for the two teachers in the research, changes in time resulted in changes in their interactions with the environment in this study.

## Methodology

### Participants

This research aimed to investigate the emotional development of Chinese high school mathematics teachers who participated in a teaching improvement program. The research object was selected using the principle of purposeful sampling to identify information-rich cases to analyze and illuminate the research questions (Patton, 2002, p. 230). In this study, two teachers, Ms. A and Mr. B, from the same mathematics improvement program were selected to maximize the information collected on teachers’ emotional development in the context of an instructional improvement program and to enable a better understanding of the complex links between teachers’ emotional and environmental interactions.

Two teachers, Ms. A, with 20 years of teaching experience, and Mr. B, with 15 years of teaching experience, both in the middle and late stages of their careers, taught in two high schools in Shanghai, China. In the spring semester of 2018, a university in Shanghai organized a teaching improvement program for secondary school teachers to recruit participants. This teaching improvement program was intended to encourage high school teachers to integrate the history of mathematics into mathematics teaching, consequently improving students’ mathematical knowledge and literacy. From 2018 to 2022, the program was conducted in two phases, each lasting 2 years, and it was a nonmandatory program organized by the noneducation administration. Participants volunteered to take part in the program, and they all adopted value recognition and eagerness to integrate the history of mathematics into mathematics teaching. Thirteen senior secondary mathematics teachers joined the first phase, and this number increased to 27 in the second phase, with both A and B participating in two consecutive phases. All teachers were required to select materials from the history of mathematics provided by the program and then use them for teaching practice. Throughout the program, they prepared lessons, discussed, observed each other’s trial and formal teaching lessons, and conducted post-lesson evaluation activities. At each stage, the program mentors (university professors) and other researchers (universities’ master’s and doctoral students) supplied them with teaching materials, evaluations, and suggestions. On average, the program held one group seminar and two class observations every month, each followed by a lesson evaluation activity. The four-year program experience allowed A and B to interact closely with the program’s personnel and experience rich life events that have caused complicated emotions. A and B believed that their participation in the program had aided their teaching practice and PD, and they were eager to share their experiences and emotions from the program and to engage in the additional data collection required for this study. The principal and some researchers in this program were included as researchers in this paper. The researchers’ acquaintances with the participants enabled them to obtain more profound knowledge of their emotions and related events, facilitating the collection of more accurate and reliable data for this study (Chen, 2000, p. 140).

Based on the above considerations, the two participants experienced wealthy emotions during their long involvement in the program, and they were willing to share their emotions and the context in which they arose. We believed that they matched well with the needs of the participants in this study and that they were valuable cases worth researching.

### Research method

This study employed a case study as a research method to explore participants’ interactions with the environment and the emotions they experienced. A case study is empirical research that delves into a phenomenon within its real-world context (Yin, 2014, p. 16). It allowed the researcher to conduct an in-depth case analysis through detailed data collection over a long period (Creswell and Creswell, 2017, p. 51). For this study, teachers’ interactions with the environment and the emotions they experienced during their participation in the program were complex. The case study method was appropriate because it allowed us to explore the complex and in-depth issue of teachers’ emotional development through detailed and in-depth data collection. Therefore, the case study method is suitable for this work.

### Data sources

Throughout the four-year program, the data on the emotional development of the two teachers were diverse and rich. Semi-structured interviews, teachers’ emotion logs, researcher memos, and informal dialogues were used as the data sources for this study. All data collection methods were ethically approved and checked by the Committee on Human Research Protection of East China Normal University.

First, the two participants provided three individual emotion logs totaling over 9,000 words. In these three emotion logs, participants recorded their main experiences, the emotions that emerged, and the events that triggered emotions in the early stage (for the first year), medium stage (from the second to the third year), and late stage (for the fourth year) of their involvement in the program. These emotion logs were adapted from the teachers’ reflection logs based on their continuous recordings over the past 4 years, and as a result, the experiences of the events in them were clearer.

In-depth, semi-structured interviews were the most significant sources of data for this study. Two teachers participated in in-depth semi-structured interviews lasting 4–6 h in the form of one-on-one online meetings. The interviews were divided into two main parts: in the first part, using the emotion logs offered by the teachers as a cue, the interviewees described their emotional development experiences during the program in chronological order, and the interviewer pursued the details and emotional experiences; in the second part, the interviewees described their most salient emotional experiences during the different stages of the program and the context in which they emerged. With the participants’ permission, all interviews were audio-recorded and transcribed verbatim, yielding about 170,000 words of interview material.

Researcher memos and informal dialogues were secondary data sources primarily used for data triangulation (Denzin, 2017, p. 297) validation. During the implementation of the program, the researcher also kept records of various seminars and observations arranged for the program, and these records could be used to corroborate key events documented in the teachers’ emotion logs. In informal dialogues, the researcher communicated certain experiences in the program through face-to-face and online talks. During these discussions, the participants conveyed some of their emotions, which served as a secondary data source.

### Data analysis

**Figure 2:**
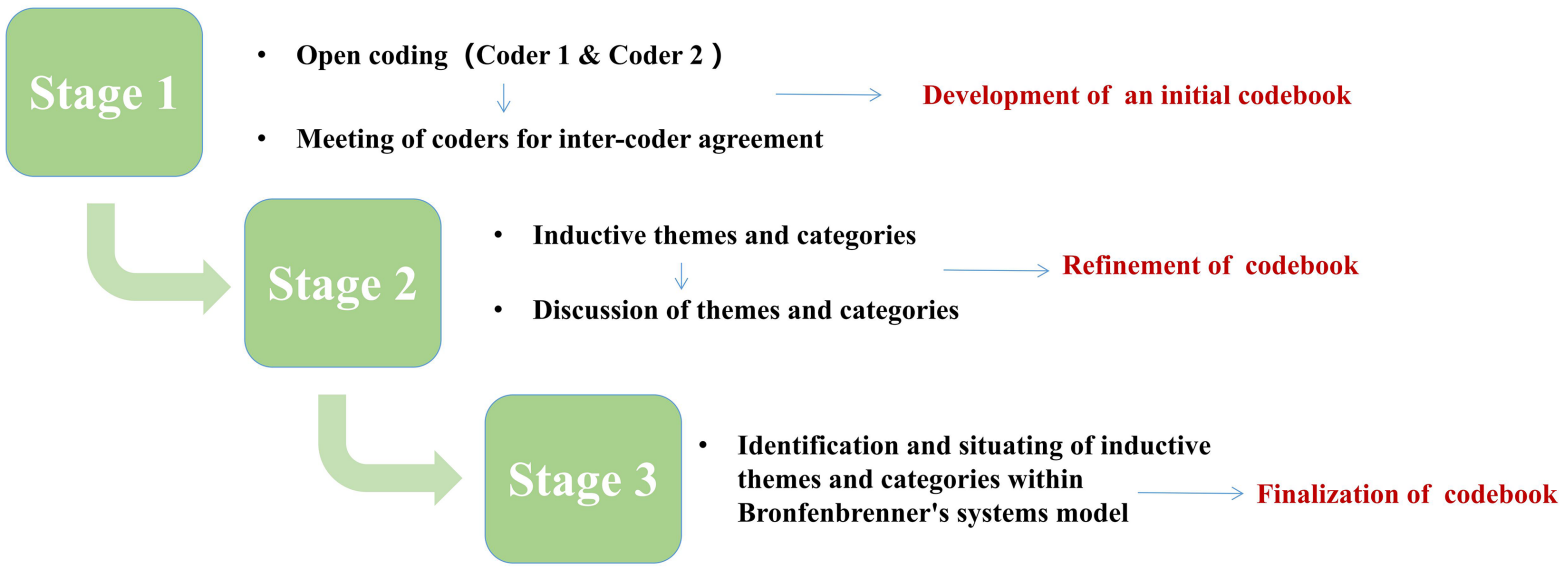
Stages of analysis implemented through applied thematic analysis.

To analyze the extensive interview data, we employed thematic analysis (Braun and Clarke, 2006) and three-step coding (Corbin and Strauss, 2014, pp. 215–310), supplemented by NVivo 12 plus as a coding tool. First, in the opening coding phase, the researcher reviewed the interview text several times and coded the sentences described by the participants regarding the individual’s interaction with the environment, the emotions that emerged, and the events that triggered the emotions, yielding 1,315 nodes (nodes are the smallest coding units of NVivo 12 plus). To establish correspondence between emotion and environment, a codebook was created to record each emotion experienced by the teachers, and the time, context, and key triggering events in the context in which it emerged, item by item, with 310 codes generated by the two teachers. Second, based on the research questions and the generalization and categorization of emotion-related terminology, the node data were classified into 68 emotions and 10 categories of environment interactive elements, and code records were added to the codebook. Finally, selective coding was employed in this study based on Bronfenbrenner’s ecological framework (Bronfenbrenner, 2005) to group the ten types of interactive elements into four systems (macrosystem, ecosystem, mesosystem, and microsystem) and to continue refining the codebook. The specific coding process could be found in the coding example Table 1 and Figure 2 depicts the steps of the complete data analysis.

**Table 1:** A sample of data coding steps.

Number	Original material	Emotion	Open coding	Axial coding	Selective coding
A-54	(Before the class) My main worry was the teaching effectiveness. Hey, nothing else was an issue. (A—The Early Stages-Interview)	Worry	Worry about the teaching effectiveness	Teaching task	Microsystem
B-107	(After the class) Our teaching and research team administrator said, “eh, this lesson is pretty good, do you want to have a special seminar?” I replied, “I’d be embarrassed to be complimented by my colleagues…” But I was also extremely proud. (B—The Late Stage-Interview)	Pride	Received compliment from teaching and research team administrator	Colleagues and administrators	Mesosystem

The highlighted section represents the teacher emotions, while the underlined section depicts the emotion’s triggering event.

### Trustworthiness

To ensure reliability in the data collection and analysis process, we first formed a data triangulation (Denzin, 2017, p. 297) validation during the data collection process, which meant that multiple data approaches were utilized to increase the internal consistency and trustworthiness of the data. Specifically, the researchers compared the teachers’ semi-structured interviews and teachers’ emotion logs with the researcher memos in detail, to verify if there were any inconsistencies. Moreover, numerous researchers conducted participatory observations on teachers’ classroom implementation, pre-session discussions, and post-session communication during the project, the results of which were recorded in the researcher memos and based on what were recorded, the investigator triangulation was formed (Denzin, 2017, p. 303). Second, an avoidance approach was employed to allow participants to express their emotions authentically during the program (i.e., the program’s principal was not involved in the data collection). At the same time, the researcher communicated informally with the participants during the data analysis process to ensure the accuracy of the emotion coding and to avoid misunderstandings. Again, two coders coded the data independently and then compared one to the other. When there was variance in the coding, a third researcher was introduced to help determine the final coding. Finally, reliability was enhanced by creating a codebook (Fereday and Muir-Cochrane, 2006), completed by all the researchers through continuous analysis, comparison, and discussion.

## Findings

Through coding and analyzing the data, the researchers revealed that 65 emotions were triggered in the two teachers’ interactions with each ecosystem over the four-year program’s participation, with 51 stated by A and 46 expressed by B. The teachers’ emotional development in different ecosystems was distinctive, and we described the teachers’ emotions and the contexts in which they emerged in each environment.

### Microsystem

Teachers who participate in the program must develop new classroom practices based on the program concepts. This style of classroom differed from that of a regular classroom and presented challenges for both teachers and students. Therefore, more frequent interactions between teachers, students, and the teaching task were formed in this classroom, eliciting diverse emotions. Among their interactions with students, the two teachers recorded 16 emotions, predominantly positive emotions and less negative emotions. Both teachers experienced complex emotions while interacting with the teaching task, describing 38 emotions.

#### Teacher–student

Regarding interactions with students in the classroom, the emotion most frequently described by the two teachers was *satisfaction*. Both teachers indicated that they had a good rapport with their students and that the teacher–student interaction was excellent. When they tried to incorporate program ideas into their classes, their students were interested, which made them feel *satisfied* and *delighted*.

Teacher B also stated that the students’ enjoyment of the teaching method gave him much *encouragement* and *firmness* in his teaching practice using the program concept.

“When I started these classes, the kids appreciated it, and to be honest, … students’ favor was one of the main drivers for me,” he continued. “Well, certainly, it is their enjoyment that keeps you going” (B-Interview).

The emotions elicited by Teacher A’s interactions with the students were more tortuous and varied. During her participation in the program, she designed two assignments on mathematical writing in which students read literature on mathematical culture and submitted short essays. In the first assignment, she was *disappointed* because the students completed the task perfunctory; in the second assignment, she was *surprised* and *moved* because the kids took the assignment seriously and were motivated by her to think deeply about mathematics.

“I was both surprised and moved,” she said. “Well, sure, because these 17 (student) essays contrasted sharply with the summer reading I had assigned a year before. After only over a year, the students could generate such a stark contrast, and their comprehension of mathematical culture and the application of mathematical history far exceeded my expectations” (A-Interview).

She was *pleased* that the students were cooperating with her more and more. This cooperation is evident not just in students’ academic performance but also in their active participation in class. She was at *ease* when her students could follow her teaching ideas. The program team occasionally arranged for her to teach unknown students, which made her *worry* about the students’ cooperation and *pity* for their lack of tacit understanding.

Finally, Teacher A stated that students often asked about her experiences after participating in the program. She *liked* the student–teacher interaction. She is also *proud* that the students admire her for her involvement in the program.

#### Teacher–teaching task

Teaching tasks are the most significant component of every teacher’s life since they engage with these tasks frequently and intimately. Teachers construct and complete teaching tasks to better practice teaching under program concepts. Both teachers experienced conflicting feelings of sadness and joy during the program.

In their interactions with the teaching tasks, the two teachers reported more frequent positive emotions in *satisfaction*, *relaxation*, *delight*, and *confidence* and more frequent negative emotions in *dissatisfaction*, *confusion*, *uneasiness*, and *stress*. The two teachers repeatedly mentioned these emotions in their interviews and emotion logs.

Positive emotions mainly came from the teachers when they accomplished their teaching tasks and performed well. Additionally, some knowledge, skills, and conceptual upgrades generated positive emotions for them during the teaching design process.

Teacher A felt *satisfied* and *delighted* after completing her first teaching task under the program concepts, which was her first official teaching attempt after 1 year of participation in the program. She felt *exhilarated* even before class, which she considered a new starting point for her PD.

Teacher A said, “It is finally a job well done. I’ve finally had a high school mathematics presentation class that integrated the history of mathematics; there is still some more *delight* in that” (A-Interview).

Positive emotions emerged in Teacher A’s teaching practice whenever she believed that she had fulfilled the established teaching objectives and that class performance was more effective. She became more familiar with teaching tasks using this method as she achieved success in completing teaching tasks and her sense of program concepts. As a result, she became more *confident* and *relaxed* in her teaching tasks and more *carefree* in completing them. Additionally, she had grown to *like* this type of teaching and become more *firm* in her mind under the program concepts.

Similarly, Teacher B’s emotions emerged. He felt *satisfaction* and *delight*, even *pride* and *love*, in accomplishing the task effectively. He also felt more *confident* and *relaxed* after gaining experience. Furthermore, he found the process of completing the task *rewarding*. Teacher B was likewise a person who continually and deeply considered the problems of the task in teaching preparation; he was *delighted* when he solved them and *satisfied* when he incorporated his unique teaching objectives into teaching design and *anticipated* his teaching effectiveness. For example, Teacher B included traditional Chinese culture in his teaching design for classical models of probability, which is his longstanding teaching aspiration. “Then this class, I think one of the highlights for myself is that I blended this traditional Chinese culture very effectively with this lesson,” he explained (B-Interview).

However, teaching practice was not always smooth. Two teachers experienced negative emotions while preparing for and implementing their teaching. They both felt *dissatisfied* with the poor teaching effectiveness, *uneasy* and *confused* because they were unfamiliar with the relevant historical material and practice methods, and *stressed* in the preparation process.

Furthermore, when Teacher A first began practicing, she frequently felt unprepared and incompetent, which made her *worried* about her teaching effectiveness. She was *cautious* and *confused* about preparing her designs and even *nervous* during class.

Teacher B was *anxious*, *fearful*, and *nervous* about the unknown teaching effectiveness when preparing the teaching design. He spent a lot of time thinking about the task to accomplish it excellently, which he discovered was *hard* and *distressing*.

### Mesosystem

The mesosystem connects two or more microsystems. The two teachers involved in the improvement program interacted in the mesosystem, primarily with the microsystems at the school and program levels.

#### School level

The school serves as both an organization and a workplace for teachers. At the school level, teachers have more frequent connections with colleagues and administrators, and these interactions can trigger their emotions.

##### Colleagues

Teachers interact most closely with their colleagues in the workplace. After participating in the program, the two teachers developed 12 emotions in their interactions with their colleagues.

Initially, the two teachers were the only ones at their schools engaging in this PD program. Their colleagues were unaware of the teaching concepts and professional knowledge of the program. However, these two teachers had relatively good mental health and did not develop too many negative emotions. The most often stated emotions were *satisfaction*, *calmness*, and *unhurried*.

Teacher A stated that each teacher held various views on mathematics education. When colleagues who did not comprehend her concepts made casual comments about her teaching attempts, she remained *calmness* and *sympathetic*. She was *indifferent* to her colleagues’ comments. Although her colleagues could not assist her PD, she was *satisfied* that her colleagues spiritually encouraged her in the program.

“It is okay if colleagues do not make a professional evaluation or comprehend my teaching concepts; it’s normal… They are willing to lend me this class, right? They are willing to devote time to supporting me. All of these are priceless to me” (A-Interview).

In addition to Teacher A’s emotional experiences, Teacher B experienced some more emotions. During the program’s medium-stage teaching attempts, Teacher B was occasionally criticized by more sophisticated teachers among his colleagues for poor teaching effectiveness, but his strong sense of identity allowed him to be *unhurried* and not eager to argue. However, his lack of communication with his colleagues made him feel *dreary*.

“It gets *dreary* at times. When you want to discuss an issue, you might not find somebody to talk to” (B-Interview).

The situation altered during the program’s late stage when Teacher B’s teaching effectiveness improved and his colleagues recognized his teaching concepts, and even younger colleagues were expected to join the program, making him feel *satisfied*, *delighted*, and more *relaxed*. He felt *proud*, especially when the colleague who had previously criticized him turned to praise him.

##### Administrators

School administrators play a crucial role at the school level, and their support and approval are essential sources of sustainability for the teachers. The two teachers’ school administrators strongly supported classroom practice under the program concepts, resulting in highly positive emotions of *satisfaction* and *rejoice* in the teachers’ interactions with the administrators.

Two teachers stated that the school administrators have been highly supportive of them since the beginning of the program and have made their teaching practice more convenient. For example, when teachers were required to present open lessons in school (which meant that many teachers from outside the school came to observe), the administrative staff were very helpful in adjusting the teachers’ schedules, and the logistics staff provided them with space. All of these made them feel *satisfied*.

“Our president was a special-grade math teacher,” Teacher B added. “So, even though we were busy then, he was supportive of us (in the program)… The president has a unique perspective in these fields… I believe I am still quite *rejoiced*” (B-Interview).

He felt *rejoiced* to have such a president and felt *satisfied* when the president praised him for his progress in teaching.

#### Program level

Once the teachers were involved in the teaching improvement program, they interacted with each other more frequently by talking and discussing their classrooms with others in the program. Both teachers generated rich emotions from their interactions with the program mentors and their program companions.

##### Program mentors

Two teachers generated 11 emotions during their long-term interactions with the program mentors.

*Admiration* was a common emotion described by the two teachers. They *admired* the program mentors’ extensive knowledge and high-quality teaching guidance. At the same time, Teacher B *rejoiced* to have met the team mentors and accepted their guidance. “We could meet a lot of big names in person, could not we?” Teacher B explained, “For example, I said we got to meet the living Professor P, right? We get to meet the celebrity in the flesh, unlike others who can only read articles (and never meet the professor in person), right?” (B-Interview). The mentor’s authority in the history and pedagogy of mathematics also made him more *unhurried* in conducting his teaching practice.

In contrast, Teacher A’s emotions were enriched by her interactions with the mentor. To begin with, Teacher A felt her poor professional level, which made her *uneasy* in her interactions with the mentor. However, the mentor’s patient guidance and approachable manner made her feel *delighted* and *flattered*.

Whenever a mentor guided Teacher A’s teaching design, she felt more *confident*. Over time, she was often *encouraged* and *reassured* about her classroom practice. It is worth noting that she was occasionally resistant to r*ejection* when she disagreed with her mentors. She gave an example of an exchange in which “the two mentors gave me this one piece of advice. At the time, I was quite *rejected* because I thought it did not seem to have much relevance to my secondary school teaching” (A-Interview).

##### Program companions

During their participation in the program, the two teachers interacted most with their program companions. During the four-year program participation, the two teachers and their program companions studied teaching materials, performed teaching seminars, and observed mathematics classrooms together, making memories and evoking many emotions—a total of 36. The most frequently reported positive emotions were *delight*, *admiration*, *like*, and *excitement*, whereas negative emotions were less frequent, comprising mainly *pity*, *stress*, and *self-abasement*. The two teachers highlighted other, more neutral emotions, such as *amazement* and *thrill*.

During their interactions with their program companions, the two teachers were most moved by *admiration* and *delight* emotions. Two teachers *admired* the sophisticated mathematics teachers’ wealth of teaching experience and knowledge. At the same time, interacting with these excellent program companions made Teacher A feel *excited*, and Teacher B was *rewarded*. When some expert teachers approved the team companions, Teacher A was particularly *excited*, and Teacher B was *delighted*.

During the program, the teachers observed and discussed each other’s classrooms. When observing classes, they were often *amazed* by the ingenious teaching design and splendid class effectiveness of their companion’s classroom design, as well as *delighted* and *admired* by the class observation process. Teachers A and B mentioned their companions who had only been teaching for 2 years at a class observation. The colleague’s class was very creative and had an active atmosphere. The students’ feedback was interesting. It was *newness* for B, and the two teachers were *thrilled* and *liked* the creative teaching method. They also gained *confidence* and *encouragement* from their young companion’s successful teaching experiences.

“I was *amazed* to observe his class because he was so good. He’s so young and so at ease in the classroom that you’d never guess he’d actually just graduated” (A-Interview).

In addition, many of the program companions formed great friendships over time, and Teachers A and B drew close to other like-minded program companions, which triggered tender emotions in them. Teacher A said the friendship made her feel *delighted*, *comfortable*, and *moved*. As a result of this like-minded exchange, Teacher B was more *firm* in the program. He was particularly *interested* in his program companions’ equal and unfettered communication. He was particularly *amazed* and *moved* by the fact that during his first teaching practice with the program concepts, one of his Ph.D. companions traveled a long way to his school to listen to and interact with him. Teacher B said in his emotion logs, “I was quite moved by the fact that Dr. X traveled a long way to listen to my lesson during the trial lecture” (B-Reflective Report).

During the process, some negative emotions may occur. The teachers may experience *self-abasement* due to their companions’ excellence, or they may experience *stress* when observing their exceptional mathematics classes. When they do not receive a response to a teaching query from their companions in a seminar, they may feel *pity*.

Overall, the program companions’ amicable and friendly connections have left both teachers with many excellent and profound memories as well as positive emotions from their four-year program participation.

### Exosystem

In the exosystem, teachers have direct or indirect connections with people or organizations outside the school and the program, and these groups in the exosystem can also trigger teachers’ emotions. For both teachers, parents, other peers, and the district’s teaching and research department in the exosystem had an impact on their emotions.

#### Parents

The two teachers had little minimal direct interaction with their students’ parents and mostly interacted with them through their children. The two teachers generated only three emotions in their interactions with the parent community: *delight*, *firmness*, and *encouragement*.

Specifically, Teacher A said that she always had a wonderful rapport with the students’ parents, who were of a similar age to her and shared many of the same ideas, allowing them to communicate as friends. Aside from teaching, they discussed life topics. This mode of interaction made her feel *delighted*. This trust from the parents also enabled her to make daring teaching attempts.

Teacher B’s teaching skills were outstanding at school, and he was very popular with the parent community. Many parents asked their children to be in his class. Teacher B did not describe the emotions that came with this parental love, but his tone suggested that he was *delighted* about it.

Teacher B mentioned that during his program participation, his teaching style became more recognized and liked by parents and that this recognition *encouraged* him to remain with these teaching concepts, giving him a greater sense of *firmness* in this practice. “The students enjoyed my lesson,” he said, “and parents believed my classes were fairly good based on their children’s feedback” (B-Interview).

#### Other peers

When executing teaching practice under the program concepts, the teachers were occasionally influenced by peers outside of the school and program, which led to emotional fluctuation. Fourteen emotions were developed by the two teachers in their interactions with other peers, with the more frequently reported emotions being *unhurried*, *delighted*, *unstrained*, and *confident*.

Positive emotions were primarily derived from peer support and positive comments. When preparing their teaching designs, the teachers occasionally discussed teaching issues with other teachers outside the program and school. Moreover, with the assistance of sophisticated peers, they felt more *confident* in the classroom. Teacher A expressed *gratitude* and *delight* for their peers’ selfless assistance. She also *admired* her sophisticated peers. She said, “So, I have more admiration for him, and the counsel he provided me was precious” (A-Interview). Teacher B indicated that he was more *unhurried* and *firm* with their support.

However, the two teachers received negative feedback from their peers. Like their colleagues at school, many of their peers were unaware of the program’s teaching concepts, and some sophisticated teachers among their peers criticized them, either directly or indirectly. In this peer environment, they felt *stressful* when presenting their classrooms to their peers.

However, after a long time of practice, they were more assertive on the inside and were not bothered by their peers’ opinions. When confronted with the question and puzzle, they could remain *unhurried*. When faced with queries, they were *unstrained*. Teacher B explained, “When someone presents a query, I hold on to my opinion” (B-Interview).

#### District education administration

In China, each administrative district includes an education department, often referred to as a “teaching and research section,” and the professionals in charge of teaching and research in the discipline are known as “teaching and research councilors.” The local teaching and research section also influences teachers’ teaching practices, which might trigger teachers’ emotions. In this study, the two teachers developed six emotions that arose from their interactions with the section: *satisfaction*, *rejoice*, *dissatisfaction*, *moving*, *excitement*, and *nervous*.

The two teachers stated that the district teaching and research section was supportive of teachers’ participation in various teaching improvement programs, and the teaching and research councilors were very positive about their participation in this program and the program mentors. As a result, when they were practicing teaching under the program concepts, the district teaching and research section provided them with many opportunities to offer open lessons at the district level. The two teachers felt *satisfied* with this support.

Teacher A had more to say about this than about satisfaction. When it came time to deliver a district-level open lesson, Teacher A was *nervous* and *excited*. Teacher A’s school had never had the opportunity to conduct district-level activities prior to joining this program. She was *moved* by the teaching and research councilors’ encouragement of her teaching practice. She felt *rejoiced* in the district’s teaching and research model, which encouraged teacher development. She noted, “The other teachers would have their research directions” (A-Interview).

Conversely, Teacher B believed that the teaching and research activities guided by the district education and research section were too formal and did not enhance his teaching practice; thus, he felt *dissatisfied* with this model.

### Macrosystem

Social, cultural, and political factors in the macrosystem influence teachers’ teaching and thus affect their emotions. The two teachers produced the least number of emotions (4) in the macrosystem: *pity, worry, rejoice, and satisfaction*.

Shanghai, where the two teachers work, is one of China’s most economically developed cities and one of the most culturally open and advanced places in terms of thinking. Against this societal backdrop, teaching concepts in the Shanghai region are in the vanguard of China as a whole, which is one of the reasons this teaching improvement program has taken root in Shanghai. Teachers A and B *rejoiced* in a city with such cutting-edge teaching concepts.

Teacher A explained, “Because, compared to Shanghai, you can be sure that many things from other cities, including the concepts, are not as avant-garde as Shanghai” (A-Interview).

Teacher B specifically said, “I think Shanghai’s Gaokao (the National College Entrance Examinations) orientation is still relatively good … it focuses on the mathematical understanding I think is particularly good … because we can use the mathematics history material to deepen students’ understanding of the discipline” (B-Interview). Shanghai’s environment also *satisfied* him.

Teacher A also indicated that, according to the teacher’s management system, high school teachers had more liberty in teaching design than elementary school teachers (many primary school teachers in China prepare lessons in a unified way in a preparation group). She had more opportunities to experiment with different kinds of teaching, which *satisfied* her.

The negative emotions in the macrosystem are primarily the result of *Gaokao’s* pressure in China. Students’ examination results are utilized as a significant indicator for evaluation, from society to schools and from parents to school administrators, under the strain of the *Gaokao*. Even if new educational concepts are advocated locally, this test-oriented strategy makes it impossible to have a large-scale impact. When Teacher B initially began teaching under the program concepts, he was *worried* about being questioned by the outside world, and he often felt *pity* that so few other teachers around him shared his concepts.

In short, both teachers’ emotions were influenced by their environment’s cultural atmosphere, teaching concepts, evaluation orientation, and institutional environment.

### Chronosystem

**Figure 3:**
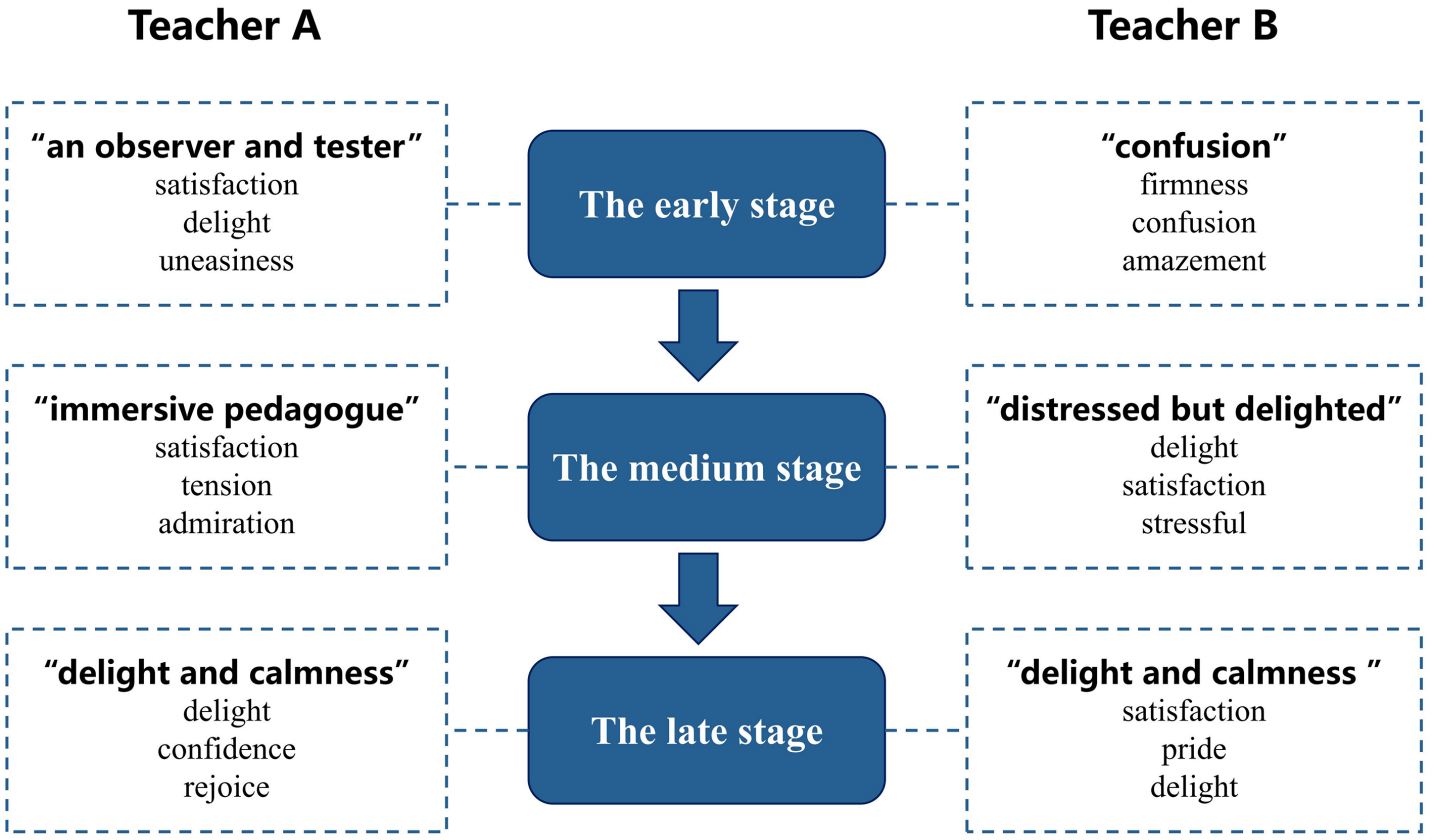
A summary graph of emotion changes of two teachers.

The teachers’ internal psychological factors and their interactions with diverse systems were altered over time. The emotions triggered by the two teachers evolved throughout the program. Figure 3 depicts their main emotions at each stage, as well as the frequently described emotions.

During the early stage of the program, two teachers were strange to the new teaching concepts and entirely unfamiliar with the teaching practice under program concepts. Teacher A characterized herself as “an observer and tester” (A- Emotion Log), experiencing positive and negative emotions. Teacher A had been participating in the program for about a year before beginning her teaching practice due to her *self-abasement*. She spent most of her time observing and learning. Her emotional resources were diverse, including teacher–student interaction and interaction with program mentors and companions, which made her *satisfied* and *delighted*. She also experienced *uneasiness* and *nervousness* due to doubts about her abilities. Teacher B used the term “confusion” (B-Interview) directly to sum up his emotions during this period. He initially believed that teaching practice under the program concepts would be simple, so he began experimenting with classroom practice as soon as he joined the program. However, while preparing for the teaching task, he found the practice difficult and felt *confused* in many areas. When his first classroom practice ended in failure, he felt *unpleasant* and *frustrated*.

During the medium stage of the program, the two teachers were actively engaged in teaching practice. However, they were not yet able to design their teaching independently and often had to seek assistance from others, and they frequently experienced emotional fluctuations due to their interactions with others. Their classroom teaching had positive and negative effects, and the emotions that were generated varied. They found the process of preparing teaching designs extremely *stressful*, but they were *delighted* when they were successful. In this regard, Teacher A described herself as an “immersive pedagogue” (A- Emotion Log), experiencing rich emotions, whereas Teacher B described himself as “distressed but delighted” (B-Interview).

During the program’s late stage, the two teachers gradually integrated the program concepts into more common classroom situations. They could primarily design their teaching independently, with few emotional fluctuations due to others’ comments. This made them feel *relaxed*, and they truly *enjoyed* the teaching process. Interestingly, Teachers A and B used the same term to describe the overall mood at this stage (i.e., “delight” and “calmness”; A-Interview, B-Interview).

## Discussion

The two teachers in this study generated emotions during program participation, which were primarily positive, with only a few interactions eliciting negative emotions. This research differs from the Sun and Yang’s (2021) study, which found that teachers in their PD programs had more negative emotions, and also differs from previous studies that discovered mathematics teachers to have more anxious emotions (Bush, 1989; García González and Sierra, 2020; Patkin and Greenstein, 2020). This was probably because the two teachers signed up for and stayed with the program for an extended period due to personal interests and PD needs. They also strongly recognized the value of integrating the history of mathematics into mathematics teaching. The love for teaching within the program concepts enabled them to maintain a positive mindset and adjust their emotions on time.

Under the guidance of program concepts, the two teachers were required to design and implement the integration of mathematics history into classroom teaching during program participation. They entered a new set of environmental systems overlaid by the program environment (see Figure 1). In the teachers’ interactions with each system, the mesosystem triggered the most emotions, followed by the microsystem, exosystem, and macrosystem, in that order. The chronosystem was employed throughout the entire program process. The majority of studies examining teacher emotions using ecosystems have indicated that teachers generate the most emotions in the microsystem (Cross and Hong, 2012; Chen, 2020). In contrast to these studies, this study focused on teachers’ interactions with program mentors and companions, which generated richer emotions and contributed significantly to mesosystem emotion triggers. This is consistent with the findings of Weddle et al. (2019), who discovered that teachers’ collaborative relationships influenced their emotions. In addition, the two teachers were part of a series of environmental systems nested in layers that influenced each other. For example, after observing their program companions’ classrooms in the mesosystem, their companions’ successful teaching gave them positive motivation, and they were more confident when faced with teaching tasks in the microsystem themselves. Conversely, when their classroom effectiveness in the microsystem was poor, they developed self-abasement in the face of program companions. These interactions between systems influenced by events are very consistent with previous research (Cross and Hong, 2012; Sun and Yang, 2021).

Indeed, once the teachers participated in the program, the emotions that arose from their interactions with the various systems influenced by the program and its concepts had some distinct features compared to previous studies. In the microsystem, when confronted with the teaching task under program concepts, the two teachers initially experienced negative emotions, such as nervousness and stress, because they were unfamiliar with the materials and the teaching model, which is in line with the existing research on the program concept’s teaching difficulties (Clark and Phillips, 2013). However, in Jiang et al.’s (2021) study on 221 high school mathematics teachers’ emotions, the emotions most frequently experienced by teachers when faced with regular teaching tasks were enjoyment and satisfaction, with only a few instances of negative emotions. It could be inferred that the negative emotions in the microsystem regarding the teaching tasks arose due to the unique difficulties of implementing teaching under the program concepts. In terms of the mesosystem, in addition to the two teachers’ unique emotional experiences at the program level, as previously described, the emotions at the school level were also distinct from previous studies. The two teachers had excellent relationships with their colleagues and administrators. Although the two teachers’ colleagues did not initially understand or approve of the program concepts, they mentally encouraged the two teachers; thus, the teachers worked positively with their administrators in transferring lessons, during which many positive emotions arose. In other studies, teachers had more challenging relationships with colleagues or administrators, and their interactions generated more negative emotions (Cross and Hong, 2012; Chen, 2020; Sun and Yang, 2021). In the exosystem, the two teachers were validated by their parents, resulting in positive emotions, and this parental support was reflected in Chen’s (2020) study of Chinese teacher emotions. However, Cross and Hong (2012) found that teacher–parent interactions frequently elicit predominantly negative emotions. Carroll et al. (2021) discovered that parental expectations could give teachers stress. These findings are dissimilar to the findings of this study. This may be because teachers in China have an “authoritative” role in the parents’ eyes, who rarely interfere with teachers’ teaching (Guo et al., 2018; Guo and Kilderry, 2018). In the macrosystem, two teachers experienced negative emotions due to the pressure of the *Gaokao* (the National College Entrance Examinations) in China, which was also reflected in studies on teacher pressure in China (Wang et al., 2009, 2015) that stemmed from China’s unique examination culture (Davey et al., 2007). Zembylas’ theory of teacher emotional genealogies might explain the commonality of the two teachers’ emotions in the macrosystem, and their negative emotions could be understood as intergroup level emotions shared in the same sociopolitical context (Zembylas, 2002). Specifically, taking the *Gaokao* as an objective in teaching is a normative practice that Chinese mathematics teachers must implement. Such practice not only disciplines the two teachers’ emotions but also makes them concerned when they are involved in teaching improvement projects. Finally, concerning the chronosystem, this study divided the two teachers’ four-year program experiences into three phases, focusing on the different emotional experiences of the teachers as a result of their personal perceptions and the environment changes over time. Other studies may also focus on teacher emotions in a chronosystem. For example, Sun and Yang (2021) studied teacher emotions at specific stages of a short-term teaching improvement program based on a narrative framework. However, this change occurred over a brief period of only 4 months and could only focus on teacher emotions at various stages of a lesson’s preparation and implementation. In contrast to this study, additional attention can be paid to the differences in emotions that arose when the same event was at different stages. Furthermore, it is worth noting that more teacher emotion-related research has ignored the chronosystem (Cross and Hong, 2012; Hofstadler et al., 2021; Liu et al., 2022).

## Conclusion and implication

In response to the research questions, this study investigated the interactions of two high school mathematics teachers in a four-year teaching improvement program with different ecosystems and the emotions that resulted from them, yielding the following findings.

First, over the four-year program, the two teachers developed 65 emotions in their interactions with the various ecosystems, with A describing 51 emotions and B describing 46 emotions. Their emotions were predominantly positive, with satisfaction, delight, and amazement being their most frequently triggered emotions. Second, regarding how these emotions were generated during the interactions with the systems, the findings revealed that the two teachers generated emotions in the microsystem mainly through their interactions with students and the teaching tasks. The two teachers triggered the most emotions in the microsystem during the preparation and execution of the teaching task. In the mesosystem, they generated the most emotions (44) through their interactions with different microsystems at the school and program levels. In particular, the richest emotions in the mesosystem were generated during learning, communication, classroom observation, and teaching evaluation with program companions, whereas the simplest emotions were generated during interactions with school administrators, with only two emotions. Interactions with parents, other peers, and the district education administration all triggered emotions in the exosystem, with peer interactions being the most significant source of emotional triggers in this system. The macrosystem, the furthest away, elicited the fewest emotions, with only four. The culture atmosphere, teaching concepts, evaluation orientation, and institutional environment all impacted these emotions. For the chronosystem, the two teachers experienced changes in their internal psychological factors and interactions with the systems during the early stage, medium stage, and late stage, resulting in significant changes in their emotions, with conflicting feelings of sadness and joy during this program and ending up in a state of delight and peace.

Based on further reflections on this study, we have gained some insights about teacher education.

First, teacher education should place a premium on developing teachers’ personal emotional competence. As the two teachers in this study became more firm and confident, they were less likely to develop negative emotions due to criticism from others. This emotional competence can assist teachers in better coping with events in their environment, reducing negative emotions and focusing on their development.

Second, teacher educators can offer a broader range of PD programs from which teachers can choose. When teachers are free to choose PD programs based on their interests and teaching concepts, they can develop more positive emotional experiences and thus be more persistent. Through the program, they are also better able to improve themselves.

Finally, teacher educators should strive to create a collaborative and communicative environment for teachers. When teachers can work positively with colleagues, companions, mentors, and administrators, they develop very positive emotions, which can motivate them to teach carefully and practice positively. Otherwise, they may generate negative emotions.

## Limitations and suggestions for further studies

This study primarily used a case study method to analyze the emotions of two teachers. The case was limited to a Shanghai high school mathematics teaching improvement program, and its findings lacked generalizability, which is a general limitation of the case study method (Yin, 2014). Second, despite data triangulation validation, the study’s primary data source was teachers’ self-reports, which may not accurately reflect teachers’ inner and actual emotions. Finally, although this study depicted the emotions generated by high school mathematics teachers’ interactions with various ecosystems’ elements, it did not go so far as to isolate the precise antecedents of emotions and establish a more direct model of causality. Therefore, in future research on teacher emotions in teaching improvement programs, the research object could be expanded and more data could be collected using questionnaires and other methods to obtain more generalizable findings. Furthermore, video recordings could be used during the program to provide a more objective view of teachers’ emotions. Finally, it is possible to investigate the antecedents that may influence high school mathematics teachers’ emotions and probable causal linkages between their emotions and specific factors.

## Data availability statement

The raw data supporting the conclusions of this article will be made available by the authors, without undue reservation.

## Ethics statement

The studies involving human participants were reviewed and approved by University Committee on Human Research Protection of East China Normal University. The patients/participants provided their written informed consent to participate in this study.

## Author contributions

PL contributed to whole design of the study, data collection and analysis, and draft writing. SH contributed to the conceptual framework, data interpretation, and draft writing. WK contributed to data collection and analysis. SL contributed to the conceptual framework. XW contributed to the conceptual framework and monitored the program. All authors contributed to the article and approved the submitted version.

## Conflict of interest

The authors declare that the research was conducted in the absence of any commercial or financial relationships that could be construed as a potential conflict of interest.

## Publisher’s note

All claims expressed in this article are solely those of the authors and do not necessarily represent those of their affiliated organizations, or those of the publisher, the editors and the reviewers. Any product that may be evaluated in this article, or claim that may be made by its manufacturer, is not guaranteed or endorsed by the publisher.
