# Supplementary material for: An ecological examination of the emotions of Chinese high school mathematics teachers in a long-term teaching improvement program
Source: Front Psychol. 2022 Dec 19;13:1033988. doi: 10.3389/fpsyg.2022.1033988 (PMC9806172; doi:10.3389/fpsyg.2022.1033988)
Supplement: Supplementary file 1 [file Table_1.docx]

Supplementary Material

# Supplementary Data

Appendix 1 summarized the concrete emotions developed by the two teachers' interactions with each system under Bronfenbrenner's ecological framework throughout the program, with emotions in each system ranked in order of frequency, and accompanied by the corresponding Chinese vocabulary.

**Appendix 1**

| **Microsystem** | **Mesosystem** | **Exosystem** | **Macrosystem** |
| --- | --- | --- | --- |
| 满意 satisfaction  开心 delight  不满意 dissatisfaction  放松 relaxation  困惑 confusion  不安 uneasiness  信心 confidence  惊讶 amazement  有压力 stressful  紧张 nervous  坚定 firmness  担心 worry  自豪 pride  平和 calmness  有收获 rewarded  放心 reassurance  感动 moving  焦虑 anxiety  喜欢 like  兴奋 exhilaration  渴望 eagerness  小心谨慎 caution  辛苦 hard  轻松 easiness  不愉快 unpleasant  爱 love  惊喜 surprise  期待 anticipated  幸运 luck  有挑战 challenging  从容 unhurried  害怕 fear  欣慰 pleasure  挫败 frustration  困难 difficulty  无动于衷 indifferent  遗憾 pity  不紧张 unstrained  畅快carefree  享受 enjoyment  激励 encouragement  失望 disappointment  痛苦 distress | 满意 satisfaction  开心 delight  惊讶 amazement  钦佩 admiration  喜欢 like  从容 unhurried  激励 encouragement  震撼 thrill  激动 excitement  庆幸 rejoice  有收获 rewarded  信心 confidence  有意思 interest  平和 calmness  遗憾 pity  坚定 firmness  有压力 stressful  赞赏 appreciation  感动 moving  自豪 pride  理解 sympathy  郁闷 depression  放松 relaxation  自卑 self-abasement  感激 gratitude  焦虑 anxiety  不紧张 unstrained  惭愧 shame  共鸣 resonance  赞叹 praise  窃喜 snigger  新奇 newness  沉闷 dreary  兴奋 exhilaration  不喜欢 dislike  放心 reassurance  受宠若惊 flattered  舒适 comfort  宽慰 relief  无动于衷 indifferent  困惑 confusion  抵触 rejection  欣慰 pleasure  不安 uneasiness | 满意 satisfaction  开心 delight  从容 unhurried  紧张 nervous  信心 confidence  坚定 firmness  不紧张 unstrained  庆幸 rejoice  感动 moving  有收获 rewarded  感激 gratitude  钦佩 admiration  自豪 pride  激励 encouragement  激动 excitement  有压力 stressful  不满意 dissatisfaction  平和 calmness  困惑 confusion | 满意satisfaction  庆幸 rejoice  担心 worry  遗憾pity |
